# Oral hygiene compliance in orthodontic patients: a randomized controlled study on the effects of a post-treatment communication

**DOI:** 10.1186/s40510-016-0154-9

**Published:** 2016-12-19

**Authors:** Mauro Cozzani, Giulia Ragazzini, Alessia Delucchi, Sabrina Mutinelli, Carlo Barreca, Daniel J. Rinchuse, Roberto Servetto, Vincenzo Piras

**Affiliations:** 1 Department of Orthodontics, School of Dental Medicine, University of Cagliari, Cagliari, Italy; 2 Via Fontevivo 21 N, La Spezia, Italy; 3 School of Dental Medicine, University of Cagliari, Cagliari, Italy; 4 51 Longlands Court, Westbourne Grove, W112QF London, UK; 5 Via della Libertà 61/30, Rapallo, Italy; 6 Via Brennero, 260/B, Trento, Italy; 7 Via Cesarea 10/1 16121, Genoa, Italy; 8 Seton Hill University Center for Orthodontics, 2900 Seminary Drive, Building E, Greensburg, PA 15601 USA; 9 Corso Galliera, 12, 16142 Genoa, Italy; 10 Via Binaghi 4/6, 09121 Cagliari, Italy

## Abstract

**Background:**

Several studies have recently demonstrated that a post-treatment communication to explain the importance of an oral hygiene can improve the orthodontic patients’ compliance over a period of 66 days. The main goal of this study is to evaluate the effects of a structured follow-up communication after orthodontic appliance application on oral hygiene compliance after 30–40 days.

**Methods:**

Eighty-four orthodontic participants enrolled from patients who were beginning fixed orthodontic treatment at the Orthodontic Department, Gaslini Hospital, Genova, between July and October 2014 were randomly assigned to one of three trial arms. Before the bonding, all patients underwent a session of oral hygiene aimed at obtaining an plaque index of “zero.” At the following orthodontic appointment, the plaque index was calculated for each patient in order to assess oral hygiene compliance. The first group served as control and did not receive any post-procedure communication, the second group received a structured text message giving reassurance, and the third group received a structured telephone call. Participants were blinded to group assignment and were not made aware that the text message or the telephone call was part of the study. (The research protocol was approved by the Italian Comitato Etico Regionale della Liguria-sezione 3^ c/o IRCCS-Istituto G. Gaslini 845/2014, and it is not registered in the trial’s register.)

**Results:**

Thirty patients were randomly assigned to the control group, 28 participants to the text message group, and 26 to the telephone group.

Participants who received a post-treatment communication reported higher level of oral hygiene compliance than participants in the control group. The plaque index was 0.3 (interquartile range (Iqr), 0.60) and 0.75 (Iqr, 1.30), respectively, with a significant difference (*P* = 0.0205).

**Conclusions:**

A follow-up procedure after orthodontic treatment may be an effective tool to increase oral hygiene compliance also over a short period.

## Background

### Oral hygiene in orthodontics

Oral hygiene is an important factor controlled by the patient during orthodontic treatment, which can affect the quality and timing of the therapy. Previous studies have demonstrated a rapid decline in oral hygiene compliance after the initial bonding, and the appliance favors plaque accumulation and represents an obstruction to the hygiene procedures [1].

Optimal oral hygiene requires thorough and clear professional instructions, adequate tools, and patient motivation, which is an essential factor to obtain compliance [2, 3]. A systematic review of randomized controlled trials conducted by Gao and colleagues [4] showed varied success in using motivational approaches, which are potentially more useful to achieve behavioral changes than conventional health education, focusing on disseminating information and giving normative advice. Studies in medicine and dentistry have asserted that active reminders induce positive behavioral change and disease prevention. A 2009 systematic review of the influence of text messages on behavior changes in the medical field demonstrated positive behavior changes in 13 of the 14 studies examined by the authors, including smoking cessation therapy, diabetes self-management, and anti-obesity behavior [5]. In dentistry, telephone calls and text messages can reduce appointment no-show rates [6–8].

A follow-up procedure, which provides encouragement and reassurance after orthodontic treatments, may be helpful in reducing the participants’ feeling of uncertainty and anxiety. The gate control theory [9], which integrates the sensory aspects of pain with cognitive behavioral and psychological factors, indicates that both anxiety and stress are directly correlated with the perception of pain that can be considered like a negative stimulus to obtain a proper behavior. Several studies have recently demonstrated that a telephone call or a text message from a healthcare provider during the first hours following orthodontic treatment significantly decreases post-procedural pain and anxiety [10–12], facilitating also health-related lifestyle changes [13], as these patients are less worried and they showed more aptitude for following oral hygiene instructions.

Bowen and colleagues [14] have shown text messaging to be an effective tool for improving oral hygiene compliance in orthodontic patients.

A post-procedure communication between the orthodontist and the patients demonstrates that the orthodontist is concerned about patient’s well-being, increasing patient’s satisfaction, and improving orthodontist-patient relation. Reinforcing reciprocal confidence, the orthodontist will obtain a better adherence to oral hygiene protocol, making patients more aware about the advantages of a correct behavior [15].

The aim of this study was to investigate in a short period of time the effects of a post-procedure communication on the oral hygiene compliance in a cohort of participants who underwent orthodontic treatments. As a secondary outcome, this research evaluated the best way to communicate with patients after the treatment to obtain the most compliance, comparing the effects of a structured telephone call to the ones of a structured text message.

## Methods

### Guidelines

**Fig. 1:**
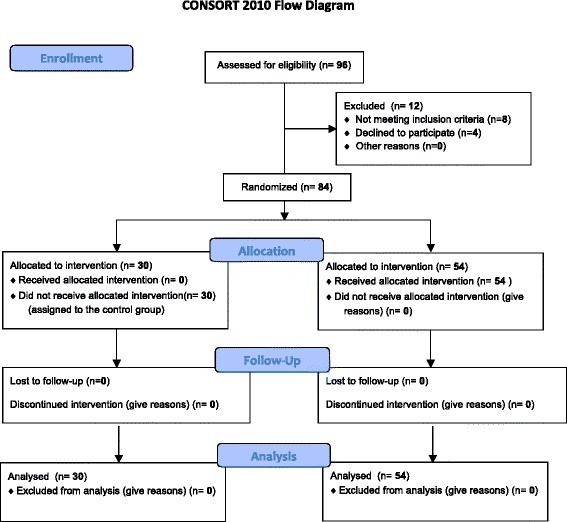
CONSORT 2010 patients’ flow diagram

The study was conducted according to current guidelines of CONSORT 2010 [16] (Fig. 1).

### Subjects, eligibility criteria, and setting

Participants were enrolled from patients who were beginning fixed orthodontic treatment at the Orthodontic Department, Gaslini Hospital, Genova, between July and October 2014. Ninety-six subjects were examined, and 88 patients fulfilled the inclusion criteria. Among these 88 patients, 84 patients and their parents consented to participate in the study. Fifteen male and 15 female participants with a mean age of 13.5 (SD, 1.7) were randomly assigned to the control group; 12 male and 16 female participants with a mean age of 12.8 (SD, 1.5), to the text message group; and 16 male and 10 female participants with a mean age of 13.6 (SD, 1.7), to the telephone group (Table 1).

**Table 1:** Descriptive statistics (ratio males/females and mean age) of the groups control, text message, and phone call

	Control	Text message	Phone call
	(*N* = 30)	(*N* = 28)	(*N* = 26)
Ratio male to female	15:15	12:16	16:10
Age			
Mean (SD), years	13.5 (1.7)	12.8 (1.5)	13.6 (1.7)
Range, years	10 to 18	10 to 16	11 to 17

Prior to participation, informed consent was obtained from all participants and from the parents of children younger than 18 years old. Inclusion criteria were participants’ age between 10 and 19 years, access to a mobile phone, orthodontic treatment with fixed maxillary self-ligating appliances, no extraction treatment, no previous orthodontic treatment, no chronic usage of analgesic medications, no previous pain-related pathology or disease, and with permanent dentition.

### Randomization method

Participants were randomly assigned to one of three intervention trial arms by means of a block randomization performed by the Department of Epidemiology and Biostatistics of IRCCS-Istituto G. Gaslini.

### Treatment protocol and process

Group 1 served as the control, and participants did not receive any kind of structured follow-up. Participants in group 2 received a structured text message offering encouragement and enquiring about participants’ well-being after their initial orthodontic treatment. Participants in group 3 received a structured telephone call after a few hours from orthodontic appliance placement. The phone calls and text message were performed 5–7 h after the bonding by the orthodontists. The structure of the call consisted of the following aspects: (i) to thank the participant for participating in the study and for attending the previous orthodontic appointment, (ii) to explain possible reasons of pain or discomfort, (iii) to encourage appropriate dental hygiene, (iv) to recommend adequate use of painkillers/analgesics, and (v) to stress the importance of a positive attitude towards orthodontic treatment.

All patients were bonded with fixed self-ligating maxillary appliances, but depending on the degree of crowding, some variation of the initial arch wire may have occurred in certain cases. This was not accounted for during the randomization process.

The participants and their caregivers in the case of youngsters were informed on the possible consequences of orthodontic treatment, such as pain, ulcers, difficulty in chewing hard food, subsequent changes in the daily diet, and remedies which could reduce discomfort. Before the bonding, all patients underwent a session of oral hygiene aimed at obtaining an plaque index of “zero” and they also received standardized instructions on oral hygiene procedures. At the following appointment, after about 30–40 days, the participants’ compliance to oral hygiene was measured using the modified Silness and Loe index, a specific orthodontic index of plaque tool [17, 18]. It was recorded by analyzing the amount of plaque circulating a periodontal probe between the bracket base and free gingival margin at six sites around each bonded tooth. The plaque index (PI) was scored as described in Table 2.

**Table 2:** Plaque index (PI) codification

0	No plaque
1	Discontinuous band of plaque at gingival margin identified just by a probe
2	Visible but moderate band of plaque at the gingival margin and at the tooth surface
3	Copious amount of plaque covering 2/3 or more of surface

One measurement was recorded for each examined site; the sum of these was divided by six to obtain the average of the plaque index for every tooth. The sum of averages was again divided by the number of total teeth.

### Objectives

The plaque index at T1 of the control group was compared to the one at T1 of the patients, who received a post-procedure communication, to evaluate if a follow-up communication after orthodontic treatment may be an effective tool to increase oral hygiene compliance.

To investigate the secondary outcome, the plaque index at T1 of the patients that received a structured text message was compared to the results of the third group, who received a structured telephone call, to demonstrate which approach is the most effective.

### Blinding

Participants were blinded to group assignment and were not made aware that the text message or the telephone call was part of the study. A blinded examiner performed data collection and analysis. A blinded examiner was calibrated for PI analysis by performing repeated measurements on orthodontic patient volunteers.

### Data analysis

The outcome (plaque index (PI)) was expressed as median and interquartile range (Iqr), because it was not normally distributed (Shapiro-Wilk test, *P* < 0.001). The estimation was run at first to compare the groups with and without a post-procedure communication (primary outcome). Then, the analysis was limited to evaluate the difference between effect of telephone call and structured text message (secondary outcome). The comparisons were performed using the two-sample Wilcoxon rank-sum (Mann–Whitney) test.

The α level was fixed at 0.05 in the primary outcome. For the secondary outcome, it was decreased to the threshold of 0.0167 for detecting statistically significant results after adjusting for subgroup multiple comparison analysis (Bonferroni correction).

The sample size was estimated a priori assuming a PI difference of 0.64 as reported from Eppright et al. [15] between the two groups of patients receiving a post-procedure communication and controls. The sample size needed amounted to 90 individuals, (SD = 1.0; α level = 0.05; power = 0.8; N1/N2 = 2/1), divided into 60 subjects for the communication group and 30 for the control group.

The statistician, who performed all the statistical analyses, was blinded to the name of the participants and the group assignment.

Statistical analysis was run using STATA 14 (StataCorp LP, College Station, TX).

## Results

### Post-procedure communication group vs control group

**Fig. 2:**
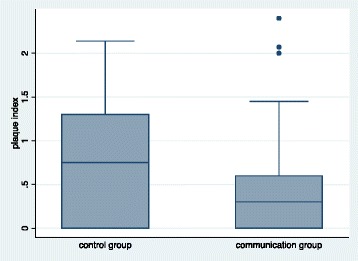
Box plot of plaque index in the control group and the communication group

Patients, who were contacted after bonding by means of a short text message or a telephone call (communication group), showed a plaque index lower than patients without contact after bonding (control group). In fact, it was 0.3 (Iqr, 0.60) and 0.75 (Iqr, 1.30), respectively, with a significant difference (Wilcoxon rank-sum, *P* = 0.0205; Table 3, Fig. 2).

**Table 3:** Plaque index (PI) change recorded at the first orthodontic checkup 1 month after professional cleaning and prophylaxis instruction

	Communication group (*n* = 28 + *n* = 26)	Control group (*n* = 30)	Comparison (*P*)^a^
Plaque index			
Median, (Iqr)	0.3 (0.60)	0.75 (1.30)	0.0205

*Iqr* interquartile range

^a^Two-sample Wilcoxon rank-sum (Mann–Whitney) test

### Text message group vs telephone call group

The difference between the text message group and the telephone call group on oral hygiene compliance (secondary outcome) did not reach the minimum α level of 0.0167 (Table 4) requested for subgroup analysis. The plaque index was 0.65 (Iqr, 0.60) in the text message group and 0.34 (Iqr, 0.23) in the telephone call group (Wilcoxon rank-sum, *P* = 0.5980).

**Table 4:** Plaque index change recorded at the first orthodontic checkup 1 month after professional cleaning and instruction in the telephone call and text message groups

	Group phone call (*n* = 26)	Group text message (*n* = 28)	Comparison (*P*)^a^
Plaque index			
Median,(Iqr)	0.23 (0.60)	0.34 (0.65)	0.598

*Iqr* interquartile range

^a^Two-sample Wilcoxon rank-sum (Mann–Whitney) test

## Discussion

### The plaque index

We analyzed the effects of two structured orthodontic follow-up procedures, a telephone call or a text message, on oral hygiene compliance after the application of orthodontic appliance.

We noticed that the oral hygiene compliance, measured by the mean plaque index, was lower in the control group compared with the patients who received a post-treatment communication.

The oral hygiene compliance of groups 2 (text message) and 3 (telephone call) showed a non-statistically significant difference. However, this last analysis was a subgroup analysis with multiplicity problems. To really evaluate the difference between the two communication methods, a new research is needed. In fact, it cannot be excluded that the effect of a communication with a text message with respect to that of a telephone call can be different. The difference also in median values of the plaque index, together with the interquartile range amount, between the two groups could raise some doubts about a real difference in influence on oral hygiene between the two communication procedures.

On the other hand, in this study design, the two methods were combined into one group only for a primary evaluation, because they are both commonly used in daily practice.

The theory of token economy [19] maintains that both positive and negative reinforcements are essential to obtain a proper behavior and to avoid the extinction process, described by Skinner [20]. The lack of compliance may be explained by the pain associated with orthodontic procedures and by the absence of adequate encouragement. We believe that post-procedure interventions may facilitate health-related lifestyle changes, reducing the individual perception of pain (negative stimulus) and promote correct habits (positive stimulus). Significant correlation has been demonstrated between psychological factors and individual perception of pain, which can influence negatively the compliance, as patients worried about pain are less inclined to follow the correct rules. The positive stimulus is represented by the promotion of correct habits, encouraging appropriate dental hygiene and stressing the importance of a positive attitude towards orthodontic treatment.

In contrast to the social psychology literature, it takes a median time of 66 days to have significant results and to turn a behavior into an automatic habit [21, 22]. In our study, differences in oral hygiene measures were seen at T1, which was two appointments after baseline. Instead, Eppright et al. [15] demonstrated significantly lower plaque index scores in the text message group just at T2, which was four appointments or an average of 5.44 months after baseline (T0). Our results show that a text message, used as an active reminder, can induce positive behavioral change also after a short period of time.

Psychological approaches, such as the cognitive restructuring method, which provide encouragement and reassurance to reduce patients’ anxiety and feeling of uncertainty, may have a positive impact on the individual perception of pain [10–12]. In our previous study, a verbal communication approach (i.e., spoken words) proved to be more powerful than a written approach on the perception of pain. Through a telephone call, it is possible to demonstrate empathy, understanding, and sharing other people’s emotions and feelings. Engaged communication has been linked to decreasing patient anxiety that can lead to physiological effects and improved outcomes [23].

Studies in medicine and dentistry have shown text messaging to be an effective tool for behavioral change; for this purpose, the emotional component of the communication style appears to be less important than the control of the perception of pain, and the easy use and the accessibility of this kind of communication appear to be the key of its effectiveness.

Since its creation over 20 years ago, SMS or short message service has revolutionized the way we communicate. In 2011, 7.8 trillion SMS messages were sent globally, highlighting that SMS is a mass communications medium used by billions of people around the globe [24].

There is a vast body of research aimed at understanding how and why people, in particular teenagers, have adopted SMS communication in their daily lives. Using both quantitative and qualitative approaches, researchers have explored various factors behind the adoption, usage, and language of SMS messages across different countries and demographics [25–28]. The studies describe that SMS or text messaging is an area of growth in the communications field. The findings suggest that text messaging is being used by a wide range of people for all kinds of activities and that for some people it is the preferred way of communication. The SMS message goes directly to the individual without external interferences, it avoids the embarrassment of a face-to-face interaction, it is more discreet than a telephone call, it gives more time to think about the content and the structure of the communication, and it guarantees the possibility to send a standard message avoiding the emotional and empathic influence of a verbal confront.

### Limitations

A limitation of the present study is that the plaque index was measured only at the first appointment after bonding. Further investigations should analyze the effects of repeated motivation on PI score trends during the entire duration of orthodontic treatment in order to provide an orthodontic hygiene protocol [2].

Even though a certain degree of bias exists in any randomized clinical trial, we tried to minimize major potential biases. In particular, an independent statistician who was not aware of the name of the participants and group assignment analyzed all our results.

## Conclusions

Our study demonstrated that regarding oral hygiene compliance, the difference between the control group and the other two intervention groups was significant.

We believe that patient motivation and reassurance, guaranteed by a post-procedural communication, are crucial factors to improve the oral hygiene compliance, also short term.
